# Occurrence of Grapevine Leafroll-Associated Virus Complex in Napa Valley

**DOI:** 10.1371/journal.pone.0026227

**Published:** 2011-10-19

**Authors:** Abhineet M. Sharma, Jinbo Wang, Siobain Duffy, Siming Zhang, Michelle K. Wong, Arash Rashed, Monica L. Cooper, Kent M. Daane, Rodrigo P. P. Almeida

**Affiliations:** 1 Department of Environmental Science, Policy and Management. University of California, Berkeley, California, United States of America; 2 Department of Ecology, Evolution and Natural Resources, School of Environmental and Biological Sciences, Rutgers University, New Brunswick, New Jersey, United States of America; 3 Texas A&M AgriLife Research, Amarillo, Texas, United States of America; 4 Viticulture Farm Advisor and County Director, University of California, Cooperative Extension, Napa, California, United States of America; Institute of Infectious Disease and Molecular Medicine, South Africa

## Abstract

Grapevine leafroll disease (GLD) is caused by a complex of several virus species (grapevine leafroll-associated viruses, GLRaV) in the family *Closteroviridae*. Because of its increasing importance, it is critical to determine which species of GLRaV is predominant in each region where this disease is occurring. A structured sampling design, utilizing a combination of RT-PCR based testing and sequencing methods, was used to survey GLRaVs in Napa Valley (California, USA) vineyards (n = 36). Of the 216 samples tested for GLRaV-1, -2, -3, -4, -5, and -9, 62% (n = 134) were GLRaV positive. Of the positives, 81% (n = 109) were single infections with GLRaV-3, followed by GLRaV-2 (4%, n = 5), while the remaining samples (15%, n = 20) were mixed infections of GLRaV-3 with GLRaV-1, 2, 4, or 9. Additionally, 468 samples were tested for genetic variants of GLRaV-3, and of the 65% (n = 306) of samples positive for GLRaV-3, 22% were infected with multiple GLRaV-3 variants. Phylogenetic analysis utilizing sequence data from the single infection GLRaV-3 samples produced seven well-supported GLRaV-3 variants, of which three represented 71% of all GLRaV-3 positive samples in Napa Valley. Furthermore, two novel variants, which grouped with a divergent isolate from New Zealand (NZ-1), were identified, and these variants comprised 6% of all positive GLRaV-3 samples. Spatial analyses showed that GLRaV-3a, 3b, and 3c were not homogeneously distributed across Napa Valley. Overall, 86% of all blocks (n = 31) were positive for GLRaVs and 90% of positive blocks (n = 28) had two or more GLRaV-3 variants, suggesting complex disease dynamics that might include multiple insect-mediated introduction events.

## Introduction

The successful management and control of plant diseases is predicated on knowledge of disease etiology and epidemiology. Although the identification of disease etiological agents has been facilitated by technological advances, notably molecular tools, there are still economically important plant diseases for which causal agents have not been conclusively identified. In addition, there are groups of pathogens that cause similar symptoms in plants and, as a consequence, the same outwardly apparent disease symptoms may have different etiological agents. Grapevine leafroll disease (GLD) is an example of such a system, where distinct virus species in the family *Closteroviridae* cause similar disease symptoms [1].

GLD was first described over a century ago and was eventually shown to be of viral etiology through assays that included graft-transmission [2]. The disease is present in all grape-growing regions of the world, including Europe, South and North America, Middle East, Africa and Oceania [3], [4], [5], [6], [7], [8], [9], [10]. The cosmopolitan distribution of GLD is likely a consequence of the movement of infected plant material. Typical symptoms of GLD include downward rolling of leaves, reddening of leaves in red grape varieties, chlorosis in white grape varieties, and limited root growth [11]. In addition to visual plant symptoms, GLD causes production impacts such as reduced yield, poor maturation of berries, low brix content in the fruit juice, and reduced wine pigmentation [11]. Graft incompatibility and other symptoms have also been associated with GLD [12]. GLD is caused by a complex of about ten virus species (*Grapevine leafroll-associated virus 1*, *2*, and so on) in the family *Closteroviridae*. Of those, most species are ampeloviruses, one is a closterovirus (GLRaV-2), and another (GLRaV-7) remains unassigned to a genus [1], [6]. Although it is known that GLD symptoms may vary based on grape variety, rootstock, and virus strain, symptoms caused by all these viruses are still grouped as one disease. However, within-species diversity, as shown for GLRaV-2, can also lead to different combinations of symptoms being expressed [12]. This is of practical relevance as management of diseases with different etiology may vary substantially. For example, because mealybugs (Hemiptera, Pseudococcidae) and soft scales (Hemiptera, Coccidae) transmit GLRaV-3, vector management is a component of disease control practices [3]. On the other hand, to date no vector has been identified for GLRaV-2 [13] and there is no evidence of this virus spreading in vineyards. Clearly, there is great insight to be gained from identifying the major causal agents of GLD in specific regions.

Surveys of GLRaVs in different regions of the world have shown that these viruses are widespread, that multiple species are present in the same region and vineyard, and that mixed infections in single plants are frequent [4], [8], [9], [10]. In general, surveys use ELISA-based approaches and these tests are occasionally followed by RT-PCR based detection methods, especially in situations where antibodies are not available for a specific virus species. However, testing large numbers of samples using RT-PCR based molecular tools is often cost prohibitive. Additionally, both approaches allow for species-level diagnosis of these viruses, but do not offer detailed data on the genetic structure of these pathogens. Nevertheless, evidence that GLRaV-3 is the etiological agent of GLD linked to epidemics in New Zealand, South Africa and Europe, and the fact that this species is readily transmitted by mealybugs, has facilitated the development of disease control strategies in those countries [3], [14], [15]. In the United States GLD is also present in all major grape-producing regions. In California, GLRaV-3 incidence was reported to be increasing annually in a vineyard in Napa Valley and several hypotheses have been proposed to explain the apparent increase in GLD incidence in the Napa Valley, including introduction of a new species/variant of GLRaV, changes in rootstock, potential vector populations, and new horticultural practices [16]. Ultimately, a combination of the aforementioned factors has brought attention to this problem in the Napa Valley.

The aim of this study was to determine which GLRaV species is most commonly associated with GLD spread in the Napa Valley by focusing specifically on vineyards with evidence of disease symptoms. A hierarchical testing structure was used in which plant samples were tested for six GLRaV species. A similar approach was repeated for different variants of the most common species identified. This sampling and testing structure was adapted to generate much needed information on the GLRaV species present across Napa Valley. Ultimately, the resulting data might provide insight into whether a specific GRLaV species, or variant, is driving the perceived GLD epidemic in Napa Valley.

## Methods

### Sampling structure

A total of thirty-six blocks with GLD symptomatic plants were sampled in October and November 2009 (Table S1). Block 7 tested positive for GLRaV-3 during an earlier survey for GLRaV-3 and was subsequently used as a positive field control. Additionally, sequencing data was obtained from this site to complement the final analysis. The blocks were distributed across 11 different vineyards in six different regions (appellations) of Napa Valley. Within each vineyard, blocks were usually located adjacent to each other (9 of 11 vineyards); the use of block pairs would allow for inferences on disease spread. The blocks were selected based on the presence of foliar GLD symptoms, grower-provided information indicative of recent disease spread, and personal observations by the authors on spatial patterns of disease occurrence in the field (i.e. disease gradient in young planting decreasing with distance from adjacent older block). Vineyard blocks knowingly established with virus-infected plant material were not included in the study. Vineyards established with certified, clean propagative material were preferentially sampled; information was not confirmed experimentally by testing clones used to establish these vineyards. Detailed information for each block is provided in Table S1. Each sample consisted of one petiole from a symptomatic plant leaf and each sample was labeled with a block number followed by a sample number within the block (e.g., 25-4 was the fourth sample tested in block 25). Studies have shown that GLD epidemics grow slowly [14], [17], [18]. Thus, performing typing studies to identify the species of GLRaV currently spreading in the Napa Valley on sites with quantitative evidence of disease spread would take several years.

### RNA extraction

Petioles were stored at −80°C and RNA extractions were eventually performed from 100 mg petiole samples using a modified version of the protocol described by Osman et al. [19]. Each petiole was cut with a razor blade into small pieces and placed into a 2.0 ml microcentrifuge tube with a pre-sterilized 1/8th inch chrome ball bearing (Boca Bearings, Delray Beach, FL); 1.8 ml of extraction buffer (1.59 g/l Na_2_CO_3_, 2.93 g/l NaHCO_3_, pH 9.6 containing 2% PVP-40, 0.2% bovine serum albumin, 0.05% Tween 20 and 1% Na_2_S_2_O_5_) was added to each tube. Samples were macerated using a Precellys 24 Tissue Homogenizer (Bertin Technologies, Catalog 03119.200.RD000) run at 6,500 Hz for two 10 sec cycles with a 30 sec intermission between cycles as suggested by the manufacturer. Following maceration, samples were centrifuged for 3 min at 16,000 rpm and 1.5 ml of supernatant was stored in a new microcentrifuge tube. Positive controls for all GLRaV species tested for in this study were provided by the Foundation Plant Services (FPS) at the University of California, Davis and propagated in the Oxford Tract facility at the University of California, Berkeley.

### Species-level survey

Five to ten random samples from each block were initially tested for the presence of GLRaV 1–5 and 9. A multiplex RT-PCR approach utilizing a modified version of the protocol described by Osman et al. [19] and the fluorescently-labeled versions of the primers used in their study were used with 5′ fluorophore modifications (Table S2). Comparison assays, utilizing positive controls from our group and those provided by FPS showed that multiplexing these primer sets did not affect the detection efficiency of any GLRaV species. Two reactions per sample were prepared using a Qiagen 1-Step RT-PCR Kit (Qiagen, Germantown, MD, Catalog Number: 210212) following manufacturer's instructions and a final primer concentration of 400 nM per primer set per reaction. The primer sets included in each reaction are listed in Table S2. A third reaction used the *Vitis vinifera 18S rRNA* gene as an internal control. Due to high transcription of the endogenous *18S rRNA* gene, a final primer concentration of 200 nM was used. Samples were processed using an initial 50°C for 30 min reverse transcription step and then at 95°C for 15 min PCR activation step. Following PCR activation, 35 cycles of PCR were carried out at 94°C for 30 sec (denaturing), 56°C for 30 sec (annealing), 72°C for 1 min (extension). After a final extension at 72°C for 2 min, samples were held at 4°C and then stored at −20°C. Subsequent PCR reactions were performed under the same conditions.

The PCR product was analyzed by fragment analysis by adding 0.7 µl of PCR product to 9.7 µl HiDi formamide (Applied Biosystems, Catalog Number: 4311320) and 0.3 µl Genescan 500 LIZ size standard (Applied Biosystems, Catalog number: 4322682). Fragment analysis was performed on an Applied Biosystem's 3730×l DNA Analyzer. Results were analyzed using Applied Biosystem Peak Scanner software (ver. 1.0). Due to an occasional but noticeable signal leakage that occurred between adjacent wells because of the high sensitivity of the detection method, a conservative peak height baseline of 4,000 of the correct size and fluorophore was established as being considered positive for respective GLRaV species; empty wells that contained just formamide and size standard were used as controls. Samples with amplicons between peak heights of 2,000 and 3,999 were tested in duplicates to ensure false positives and false negatives were avoided. Samples below peak heights of 2,000 were considered negative as re-running those samples yielded inconclusive results. This setup was used for every subsequent analysis of post-PCR products. There are potential benefits to utilizing fragment analysis over gel electrophoresis. First, it allows for the high throughput processing of 96 samples in parallel rather than single samples. While gel electrophoresis provides an estimate for amplicon size based on a known ladder, fragment analysis provides an exact fragment size based on a standard curve, derived from the internal size standard. As a result, fragment size differences of as low as 15 bps can be multiplexed in the same reaction. Furthermore, since each DNA fragment is fluorescently labeled, samples are identified as positive based on exact amplicon size and fluorescent label. This lowers the probability of false positives.

### GLRaV-3 variant typing

**Figure 1:**
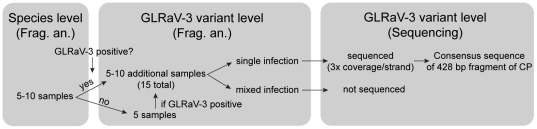
Schematic illustration of sampling design used in this study. Samples were first screened for different GLRaV species, then two approaches were used to type GLRaV-3. Frag. An. stands for ‘Fragment analysis’, a typing approach described in Materials and Methods.

Based on the results of the species level identification scheme, petioles were further tested to identify specific GLRaV-3 variants. If a block was positive for GLRaV-3, then the previous samples plus additional 5 to 10 samples were tested (Figure 1). However, if a block was negative for GLRaV-3, the five samples tested above were tested again for the presence of GLRaV-3 variants in case the primers designed for this study (described below) detected potential positives that were missed by the primer used for the species level survey. Fluorescently labeled primers (Table S3) were designed to distinguish between the four GLRaV-3 variants identified in Napa Valley, CA by Wang et al. [20]. The same terminology for genetic variants was used in this study. Forward and reverse primers were designed based on multiple alignments that identified regions conserved in one of the four variants but with low sequence similarity in comparison to the other three variants. In order to detect isolates the variant specific primers might miss, a general GLRaV-3 primer set was designed from regions of the coat protein (CP) gene conserved within the 50 isolates in Wang et al. [20] and other sequences deposited in GenBank. This primer set is hereafter designated as CP primer set (Table S3). Primer sets were first tested and assayed individually and then in a multiplex setup using the RNA extracted from a previous project [20].

All reactions were run in a 3-plex with the first reaction containing the GLRaV-3a, GLRaV-3c and *18S rRNA* gene, while the second reaction contained GLRaV-3b, GLRaV-3d, and the CP primer sets. A final primer concentration of 500 nM was used for all primer sets, except the *18S rRNA* was run at 80 nM because of the same limitations mentioned above.

### GLRaV-3 sequencing of CP gene and phylogenetic analyses

After variant-level detection, all samples identified as either single infections or those showing only the CP amplicon through fragment analysis were sequenced on both strands (Figure 1). The same method as above was used to prepare the crude extractions for PCR. Primers for PCR, CP130F and CP580R, and nested primers for sequencing, CP210F and CP500R, were designed from conserved regions of the CP gene using the same approach described to generate variant specific primers (Table S4). Three independent reactions per sample were run with a final concentration of 500 nM each and the same thermocycler conditions as above were used. After PCR, purification and sequencing were performed at Qintarabio Inc. in Albany, CA. Only samples that provided all six reads were used. Sequences were assembled into a 428 bp consensus sequence using Vector NTI version 11 (Invitrogen, Carlsbad, CA) by overlapping the three independent reads per strand. GenBank accession numbers for deposited sequences are JF421762-JF421964.

All isolates detected only by the CP primer set, except for one, generated reads when sequenced using primer 500R but not primer 210F. These isolates were subsequently labeled GLRaV-3e and for these samples, primer 130F instead of 210F was used to sequence in the forward direction. This sample set showed no variant-specific amplicons in the fragment analysis. The lone sample that generated a forward read when sequenced with primer 210F (isolate 43-15) was thought to be a mixed infection of two previously unidentified variants. Isolate 43-15 was subsequently labeled as GLRaV-3f and internal primers that were specific for GLRaV-3f were designed to sequence the isolate specifically in both directions. The primers were designed by overlapping the forward read of 43-15's sequence of GLRaV-3f with sequences from all GLRaV-3e isolates to find a region of low similarity between GLRaV-3f and GLRaV-3e (Table S4). A 428 bp region from GLRaV-3f was generated and assembled using the same method as above. Additionally, to insure the results were not caused by our multiplexing approach, nine GLRaV-3e and one GLRaV-3f samples were re-run using the GLRaV-3 HSP70h and the CP primer set in separate reactions at 1000 nM concentrations per primer set. The samples were subsequently visualized in 2.0% agarose gel.

Phylogenetic analyses included all new sequences generated here and those of 8 representative isolates (from different variants) available in GenBank for comparative purposes. Reference isolates were: NZ-1 (EF508151), 7-1006 (JF421962), 43-15 (JF421951), 7-110 (HQ130309), GP18 (EU259806), Cl817 (EU344894), NY1 (AF037268), 9-221 (HQ130332). We also ran an additional analysis with a larger set of all deposited sequences; results were similar to the tree presented here (data not shown). Sequences were manually aligned in Se-Al (http://tree.bio.ed.ac.uk/software/seal/). Six algorithms implemented in RDP3.15 [21] were used to assess for the presence of recombinants in the data set. Maximum likelihood phylogenetic analyses were conducted in PAUP* [22], with tree-bisection-reconnection branch swapping. Models for nucleotide substitution were selected by AIC in MODELTEST [23]. Branch support was estimated with one thousand bootstrap replicates.

### Geospatial mapping of GLRaV-3 variants

For all GLRaV-3 positive sites, GPS coordinates were obtained using a Garmin etrex Legend GPS navigation system (Garmin, Olathe, KS). GPS coordinates were taken at the center of the collection sites in a location clear of aerial interference. To better illustrate the distribution of a given GLRaV-3 variant, geospatial analysis was performed in ArcMap ver. 10 (Esri, Redlands, CA) using inverse distance weighting algorithm (IDW) interpolation with default power settings and the default values for the search radius. Hot Spot Analysis (Getis-Ord GI*) was performed to determine if there were regions with high and low frequency of site with a given variant. These hot and cold spots would be statistically determined; large positive Z-scores (low p-values) indicate significant clustering of high incidence values and large negative Z-score (low p-value) indicate statistically significant clustering of low incidence values. As a caveat, the Hot Spot Analysis considers and compares each strain independently. Additionally, this analysis does not identify outliers. For example, for GLRaV-3b, site 32 is a location of high incidence with 93% of the total positives containing GLRaV-3b (dark blue on the interpolation map). However, the neighboring sites have very low GLRaV-3b frequency. This results in statistically non-significant Z-scores, for a given region and therefore no clustering of sites based on high or low GLRaV-3b incidence values occurs. Finally, the results are based on a partially biased, non-random sampling method, and should not be taken as conclusive evidence of GLRaV-3 variant distribution in Napa Valley.

## Results

### Species-level survey

The initial screening to determine which GLRaV species were present in the surveyed vineyards detected GLRaV-1, -2, -3, -4 and -9 among samples, but not GLRaV-5. Sixty-two percent of tested samples (n = 216) were positive for at least one GLRaV. Of the positives, single infections of GLRaV-3 represented 81% of the samples, while 4% were single infections of GLRaV-2, and 15% were mixed infections of GLRaV-3 with either GLRaV-1, 2, 4, or 9. Mixed infections (n = 20) with GLRaV-2 and -3 were the most common with 7.5% of all samples. GLRaV-1, -4, -9 were only detected in mixed infections with GLRaV-3 (2.3, 0.4, 3.7% respectively of all tested samples). GLRaV-3 was found in 25 of the 27 positive blocks. Overall, no virus was detected from collected samples in 22% (8 of 36) of the blocks with the methodology used for the species-level survey, although three of those were later identified GLRaV-3 positive during subsequent testing (see below).

### GLRaV-3 variant typing

To further analyze the genetic structure of GLD in Napa Valley, additional samples were used to determine the variant of GLRaV-3 dominant in the tested populations. Sixty-six percent (n = 468) of plants were positive for GLRaV-3, a similar but slightly higher proportion of samples compared to the species-level survey. The difference may be due to samples that were RT-PCR positive with the CP primer set but were not detected with the heat shock protein 70 homologue (HSP70h)-based primer set used in the species-level survey. Of all samples tested, 27% were positive for GLRaV-3a, while 13% and 31% were infected with -3b and -3c, respectively. The remaining samples were primarily mixed infections of two or more GLRaV-3 variants. Mixed infections were observed in approximately 21% of positive samples, and those were dominated by GLRaV-3c occurring with either -3a or -3b (Figure S1). Of the mixed infections, one was a triple infection of variants -3a, -3b, and -3c. Single infections by GLRaV-3a and -3c were the most prevalent, while variant -3d only occurred in four samples in mixed infections with variant -3a and was limited to block 34. For samples tested using both species- and variant-level typing (n = 216), 65.2% of samples were positive for GLRaV-3. Of those, only two samples, 21-14 and 37-2, were positive using HSP70h species-level primer-set but negative with the CP variant-level set and only 21-14 provided clean reads for sequencing. On the other hand, 6% of the samples were positive using the CP primers designed for this study but negative when using the HSP70h primers. Furthermore, when representatives of the GLRaV-3e and GLRaV-3f were re-tested using the HSP70h and CP primer sets individually (n = 10), all of the isolates were positive with the CP but not the HSP70h set through fragment analysis. Nine of those ten samples were positive for the CP primer set through gel electrophoresis while again all were negative with the HSP70h set. The difference between the fragment analysis and gel electrophoresis is most likely due to the higher sensitivity of the fragment analysis method. Additionally, the GLRaV-3e samples accounted for two of the eight potentially GLRaV free sites testing positive through the variant level testing (sites 41 and 46). The third positive site, site 27, had one positive sample that also tested positive for GLRaV-3b. The same sample yielded a sequence that grouped with GLRaV-3b but was still negative when retested using the GLRaV-3 HSP70h primer set.

### Phylogenetic analyses of GLRaV-3 CP gene

**Figure 2:**
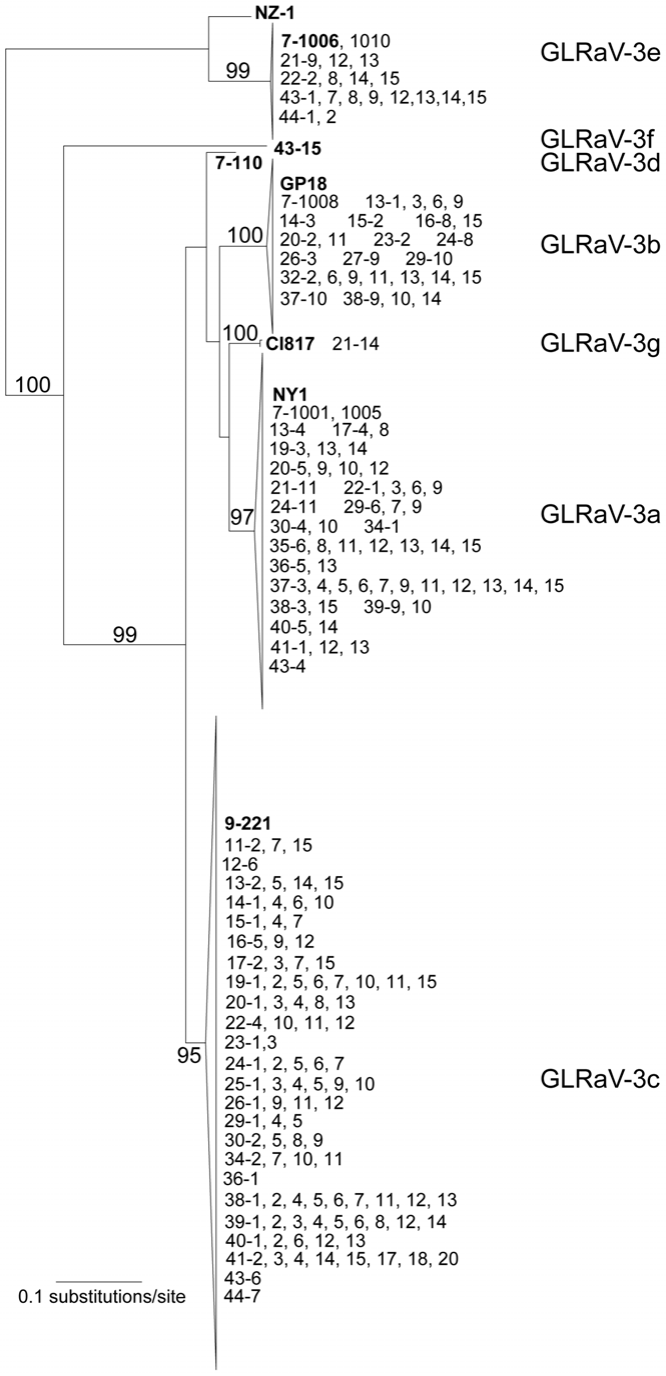
Maximum likelihood tree of a 428 bp segment of the CP gene of GLRaV-3 isolates from Napa Valley. Representative global isolates are in bold for reference. Individual sample number is listed in front of its respective block number (first number, before the dash), numbers after successive commas represent samples from the same block; longer spaces between blocks in the same line were occasionally used due to space limitations, and represent samples from other blocks. Phylogenetic clades were labeled based on Wang et al. (20) and this work. The tree is midpoint rooted for clarity of presentation and ≥70% branch support values are presented; non-supported branches were collapsed for clarity.

Seven well-supported GLRaV-3 phylogenetic clades were found in Napa Valley, in addition to isolate NZ-1 from New Zealand, which remained as the sole representative of that genetic clade (Figure 2). No evidence of recombination was found in the dataset, as previously observed for a larger fragment in the 3′ end of GLRaV-3 isolates (Wang et al. 2011). The grouping of isolates based on sequence data matched typing performed at the variant level, as mentioned above. GLRaV-3 variants may be divided into two major clades, one for which available diagnostic primer sets function well (-3a, -3c, -3d, -3b and -3g) and another for which a novel primer set (CP gene) was necessary (-3e and -3f and NZ-1; NZ-1 based on *in silico* analysis). Only one isolate of GLRaV-3f clade was found in this study, the same occurred with -3g, although that isolate grouped closely with a sequence from Chile (Cl817), which Engel et al. [24] and Wang et al. [20] found previously to belong to an independent and well supported clade. Variant -3d was previously found infecting plants in the Napa Valley [20]. Variants -3a, -3b and -3c correspond to groups I, II and III, respectively, according to Jooste et al. [25]. Additionally, for single infection plants, there was 100% concurrence between the results for the variant level typing and the results obtained from the sequencing data.

### Spatial interpolation of GLRaV-3 variants

**Figure 3:**
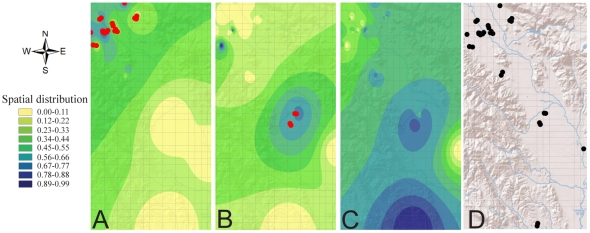
Geospatial and clustering analyses of GLRaV-3 variants in the Napa Valley. Images A through C illustrate results for GLRaV-3a, -3b and -3c, respectively; spatial distribution patterns for each variant are shown using interpolation data. The colored gradient and the corresponding values for spatial distribution represent the proportion for a given GLRaV-3 variant compared to the total number of GLRaV present in the tested block. The red dots indicate localities with statistically supported high incidence (p<0.01) of a particular variant in relationship to the remaining sample set. Image D shows all blocks positive for at least one GLRaV-3 variant. Each grid box represents 1 km^2^.

Based on the Hot-Spot Analysis, the distribution of GLRaV-3 variants in Napa Valley was variable. GLRaV-3a was more frequent in blocks in the northern section (Z>2.78, *P*<0.01), while -3b had higher prevalence in the central areas (Z>2.78, *P*<0.01). However, there were no statistically supported blocks with high or low -3c, reflecting the high frequency of -3c across the entire sample set. Figure 3 shows the geographical location of the blocks with high frequency. The interpolation data in the same figure helps illustrate the frequency of occurrence for each variant in a given area. Variants -3d and -3e were not widely distributed across the region to run the analysis, -3d was limited to one block and -3e was found in two pairs of neighboring blocks (21/22 and 43/44).

## Discussion

Surveys of plants with GLD symptoms have shown that several GLRaV species are distributed throughout grape-growing regions of the world [4], [8], [10], [26]. However, GLRaV-3 has been the species primarily associated with vector-mediated disease spread [14], [16], [18]. We found that although different GLRaV species are present in the Napa Valley, GLRaV-3 is the major species (∼80%) associated with symptomatic plant material in vineyards with evidence of recent disease spread. One tenth of positive plants were infected with GLRaV-2, which is most likely not mealybug-transmitted [13]. While it is possible that GLRaV-2 is also spreading in the Napa Valley, a more parsimonious interpretation is that the positive samples were the result of contaminated plant material. All remaining positive samples were primarily infected by GLRaV-3 and other virus species in mixed infections, highlighting the predominance of GLRaV-3 in this survey. A similar survey in New York State also found that a small proportion of vines were infected with multiple GLRaV species [4]. One issue not addressed in this study is that GLRaV-3 may reach higher within-plant populations compared to other species, thereby reducing the detection rate of the other viruses due to our multiplex approach. Although this is a possibility, the method was sensitive enough to permit for the identification of multiple species in the same sample, and to detect positive controls for all species. Other limitations of this approach are discussed more thoroughly below. Lastly, other species could have been more common if any vineyard with GLD symptoms was surveyed, as those would include blocks that were unknowingly, or knowingly, established with virus-infected plant material.

The identification of GLRaV-3 as the main species in this survey is of practical importance, as this species has been shown to be transmitted by several grape-colonizing mealybugs [13]. Furthermore, it suggests that management strategies used elsewhere may be applicable for this region. However, GLRaV-3 is subdivided into multiple variants [25], [27], [28], four of which were previously shown to occur in Napa Valley [20]. When samples were tested for different GLRaV-3 variants, seven well-supported clades of GLRaV-3 were identified based on partial CP gene nucleotide sequences. In addition, the divergent sequence of isolate NZ-1 from New Zealand formed its own clade. These results are in agreement with recent phylogenetic analyses of GLRaV-3 [20], [25], [29]. Mixed infections occurred in a representative percentage (∼20%) of positive samples in this study; a similar trend was observed in South Africa [25], where authors used a different approach for virus detection (single-strand conformation polymorphism, SSCP).

Data generated with fragment analysis largely matched sequencing data, except that sequence results have much more resolution and allowed for the detection of new genotypes. Although new variants of GLRaV-3 (i.e. supported phylogenetic clades) were found in this study, their relative frequency was lower than of those previously found in the region. Alternatively, further sequencing work for one of the new variants (-3e) showed substitutions in the region of the primer set used here (CP primer set, data not shown), which may have limited the amplification of several isolates from this population. Therefore, much in the way that three additional blocks were identified as GLRaV-3 positive when tested using CP primer set, the remaining five negative blocks (14% of total blocks) might be infected with an undetectable variant of GLRaV-3 or another GLRaV species. The same holds true for all negative samples. Using a single petiole may have been a potential limitation in the present study due to heterogeneous distribution of the virus within infected vines resulting in low population sizes in some of the sampled tissue. This might have reduced our ability to detect the virus. Regardless, these results highlight the importance and need for robust pathogen genetic diversity data for the development of molecular-based diagnostic tools since these methods are highly specific. Immunological assays (i.e. ELISA) were not used or tested during this study and their reliability needs to be confirmed. Finally, given these findings, we predict that more GLRaV-3 variants will be identified in the future.

Sequence data analyses (not shown) and phylogenetic tree topology are indicators that the GLRaV-3 variants found in this study, including the novel variants, are under purifying selection and therefore should not be considered emerging genotypes. This was also observed in a previous study in which a 4.7 kb fragment of GLRaV-3's genome was analyzed [20]. However, the fact that previously undetectable variants were detected in Napa Valley suggests that they also occur in other grape-growing regions. This is highlighted by the fact that isolate NZ-1 remains the only taxon in a divergent clade. New large-scale surveys with primer sets designed based on conserved genomic regions will assist in the identification of new variants.

Although there are no guidelines for naming GLRaV-3 variants, there are now independent terminologies being used by the community, one is based on the name of type isolates [29], another adds roman numerals to GLRaV-3 [25], and the last one adds letters [20]. We suggest that letters are more helpful than numerals because GLRaV species are already named using successive numbers [1]. The use of type isolates may have the unintended consequence of leading individuals not familiar with the system to interpret ‘variant NY-1’, for example, as originating in New York State, USA. Regardless, given that there are several well-defined genetic GLRaV-3 variants, and that these may also represent phenotypically distinct groups, it will be important for taxonomists to devise a classification method for this group of viruses.

The spatial distribution of GLRaV-3 in the Napa Valley showed that variants might be unevenly distributed in the landscape, despite the fact that mixed genotypes were found in most vineyard blocks. GLRaV-3a seems to be more concentrated in the appellations in the north (Oakville and Rutherford), while GLRaV-3b showed higher frequencies in Oak Knoll, the appellation in the center of the collection area. There were no blocks with high frequency for GLRaV-3c, supporting the fact that GLRaV-3c was found in high frequency across the entire sample set. However, a more thorough survey of the entire region is needed to draw definitive conclusions from these data. Among several factors, this survey was biased towards a limited number of vineyards with evidence of disease spread. In addition, grapevines in the Napa Valley tend to be planted following region-specific varieties based on optimal horticultural performance for wine production, and it is possible that variants of GLRaV-3 vary in their relative virulence in different host genotypes as has been previously observed with the closterovirus GLRaV-2 [12].

### Inferences on disease spread

A closer look at individual vineyards and their respective blocks was suggestive of short and long-range vector dispersal of GLRaV-3. Mixed variants were frequently found in vineyards, and two competing hypothesis explain their occurrence. First, plant material used for propagation may have been infected with multiple GLRaV-3 variants. Although this is possible, we would then expect most plants within the same vineyard to have mixed infections, which was not the case. A second hypothesis would be that multiple independent introductions mediated by vectors occurred. In this scenario, the minimum number of introductions would be the number of GLRaV-3 variants detected in a block. The maximum number is difficult to determine due to low sequence diversity within variants. A similar scenario has recently been proposed to explain GLRaV-3 spread in New Zealand [3].

The approach used here was aimed at identifying the GLRaV species spreading in Napa Valley vineyards. Despite limitations, survey results showed that the sampling design permitted inference on disease etiology and ecology without the need of multi-year surveys and provide some foundation for long-term field studies. We found that GLRaV-3 is the predominant species in vineyards with anecdotal evidence of disease spread, and that several variants within that species infected the sampled plants. Variant frequency and distribution patterns are suggestive of vector dispersal at multiple spatial scales, which if confirmed experimentally would require the establishment of local and area-wide disease control strategies. The sequence data (purifying selection) and lack of one dominant GLRaV-3 variant in the region provide no support to an epidemic-like spread of a novel GLRaV genotype. In addition, surveys on this scale may lead to the identification of previously unknown virus genotypes, which will result in better diagnostic tools.

## Supporting Information

Figure S1 **Diagrammatic summary of vineyard blocks sampled for GLRaV-3 variants.** Vineyards without positive samples or with one infected plant were not included. Each letter (A through H) represents a different vineyard, blocks' size (bars = 200 meters) and spatial location in relation to each other are accurate representations based on aerial photographs of blocks. Information per block, when available, includes block number as in Table S1, year of block establishment, and GLRaV-3 variants present in each block and the respective number of positive samples. For example, vineyard ‘C’ had two blocks surveyed, one established in 1994 (#17) and another in 2008 (#18), block #18 had no positive samples but #17 was positive for GLRaV-3a, -c, and had -3a/c mixed infections.
(DOC)

Table S1 **Information on Napa Valley vineyards and blocks surveyed for GLRaVs.** Subdivisions indicate different vineyards and the blocks therein.
(DOC)

Table S2 **Primer sets and multiplex conditions for detection of grapevine leafroll-associated viruses at the species level.** All primers were designed by Osman et al. (2007, J. Virol. Methods 141: 22–29).
(DOC)

Table S3 **Primer sets and multiplex conditions for detection of** ***Grapevine leafroll-associated virus-3*** **at the variant level.** Primer sets for the four variants diagnosed and the conserved coat protein gene (CP) primers were designed in this study but were based on work by Wang et al. (2011, Phytopathology 101: 445–450). Primer set for the internal control, 18 S, was designed by Osman et al. (2007, J. Virol. Methods 141: 22–29).
(DOC)

Table S4 **Primers used for sequencing of 428 bp of the coat protein gene of** ***Grapevine leafroll-associated virus-3*** **isolates.**
(DOC)
