# Supplementary material for: Occurrence of Grapevine Leafroll-Associated Virus Complex in Napa Valley
Source: PLoS One. 2011 Oct 19;6(10):e26227. doi: 10.1371/journal.pone.0026227 (PMC3198396; doi:10.1371/journal.pone.0026227)

**Figure S1. Diagrammatic summary of vineyard blocks sampled for GLRaV-3 variants**. Vineyards without positive samples or with one infected plant were not included. Each letter (A through H) represents a different vineyard, blocks’ size (bars = 200 meters) and spatial location in relation to each other are accurate representations based on aerial photographs of blocks. Information per block, when available, includes block number as in Table S1, year of block establishment, and GLRaV-3 variants present in each block and the respective number of positive samples. For example, vineyard ‘C’ had two blocks surveyed, one established in 1994 (#17) and another in 2008 (#18), block #18 had no positive samples but #17 was positive for GLRaV-3a, -c, and had -3a/c mixed infections.


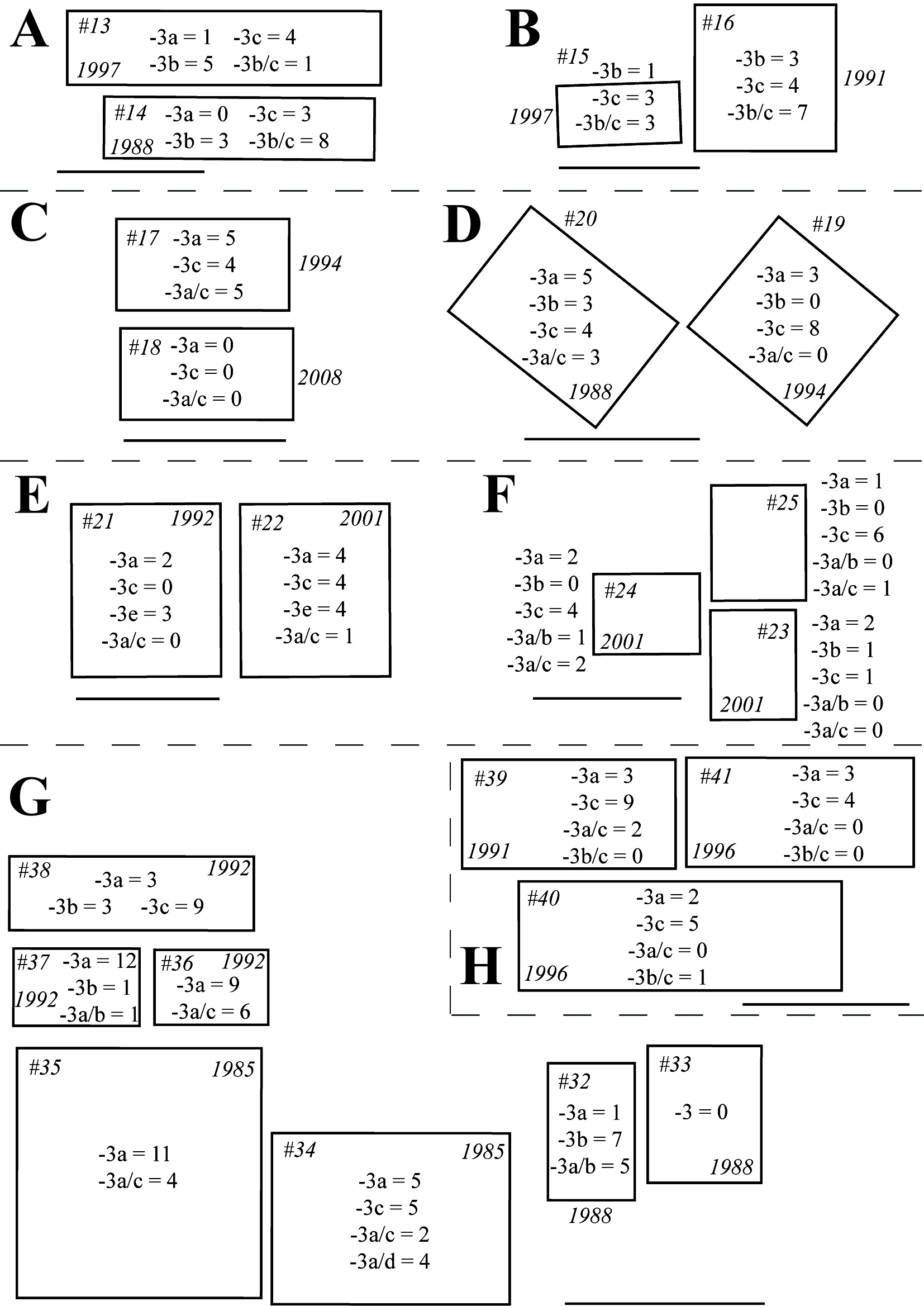

Supplement: Figure S1 — Diagrammatic summary of vineyard blocks sampled for GLRaV-3 variants. Vineyards without positive samples or with one infected plant were not included. Each letter (A through H) represents a different vineyard, blocks' size (bars = 200 meters) and spatial location in relation to each other are accurate representations based on aerial photographs of blocks. Information per block, when available, includes block number as in Table S1, year of block establishment, and GLRaV-3 variants present in each block and the respective number of positive samples. For example, vineyard ‘C’ had two blocks surveyed, one established in 1994 (#17) and another in 2008 (#18), block #18 had no positive samples but #17 was positive for GLRaV-3a, -c, and had -3a/c mixed infections. (DOC) [file pone.0026227.s001.doc]
