# Supplementary material for: Occurrence of Grapevine Leafroll-Associated Virus Complex in Napa Valley
Source: PLoS One. 2011 Oct 19;6(10):e26227. doi: 10.1371/journal.pone.0026227 (PMC3198396; doi:10.1371/journal.pone.0026227)
Supplement: Table S1 — Information on Napa Valley vineyards and blocks surveyed for GLRaVs. Subdivisions indicate different vineyards and the blocks therein. (DOC) [file pone.0026227.s002.doc]

**Table S1. Information on Napa Valley vineyards and blocks surveyed for GLRaVs.** Subdivisions indicate different vineyards and the blocks therein.

| Block # | Variety | Appellation | Adjacent to block # | Comment |
| --- | --- | --- | --- | --- |
| 7 | Merlot |  |  |  |
| 11 | Pinot Noir | Carneros | 12 | Low prevalence |
| 12 | Pinot Noir | Carneros | 11 | High prevalence |
| 13 | Pinot Noir | Oak Knoll | 14 | High prevalence |
| 14 | Chardonnay | Oak Knoll | 13 | Low prevalence |
| 15 | Cabernet Sauvignon | Oak Knoll |  | High prevalence |
| 16 | Chardonnay | Oak Knoll |  | Low prevalence |
| 17 | Cabernet Sauvignon | Rutherford | 18 | High prevalence |
| 18 | Cabernet Franc | Rutherford | 17 | Young block |
| 19 | Cabernet Franc | Yountville | 20 |  |
| 20 | Cabernet Sauvignon | Yountville | 19 | High prevalence |
| 21 | Merlot | Oakville | 22 |  |
| 22 | Merlot | Oakville | 21 |
| 23 | Cabernet Franc | Oakville | 24,25 |  |
| 24 | Merlot | Oakville | 23,25 |  |
| 25 | Merlot | Oakville | 23,24 | Edge symptomatic |
| 26 | Cabernet Sauvignon | Oakville |  |  |
| 27 | Merlot | Oakville | 28 |  |
| 28 | Malbec | Oakville | 27 |  |
| 29 | Cabernet Sauvignon | Oakville |  | High prevalence |
| 30 | Cabernet Sauvignon | Oakville |  |  |
| 31 | Merlot | St. Helena |  |  |
| 32 | Merlot | St. Helena | 33 | Edge symptomatic |
| 33 | Merlot | St. Helena | 32 | Edge symptomatic |
| 34 | Cabernet Sauvignon | St. Helena | 35 | High prevalence |
| 35 | Cabernet Sauvignon | St. Helena | 34,36,37 | High prevalence |
| 36 | Cabernet Sauvignon | St. Helena | 35,37,38 |  |
| 37 | Cabernet Sauvignon | St. Helena | 35,36,38 |
| 38 | Cabernet Sauvignon | St. Helena | 36,37 |
| 39 | Cabernet Sauvignon | St. Helena | 40,41 | High prevalence |
| 40 | Cabernet Sauvignon | St. Helena | 39 | Edge symptomatic |
| 41 | Cabernet Sauvignon | St. Helena | 39 | Edge symptomatic |
| 42 | Malbec |  |  | High prevalence |
| 43 | Merlot | Oakville | 44 | High prevalence |
| 44 | Merlot | Oakville | 43 | Edge symptomatic |
| 45 | Merlot | Oak Knoll | 46 | High prevalence |
| 46 | Merlot | Oak Knoll | 45 |  |
