# Supplementary material for: Occurrence of Grapevine Leafroll-Associated Virus Complex in Napa Valley
Source: PLoS One. 2011 Oct 19;6(10):e26227. doi: 10.1371/journal.pone.0026227 (PMC3198396; doi:10.1371/journal.pone.0026227)
Supplement: Table S2 — Primer sets and multiplex conditions for detection of grapevine leafroll-associated viruses at the species level. All primers were designed by Osman et al. (2007, J. Virol. Methods 141: 22–29). (DOC) [file pone.0026227.s003.doc]

**Table S2. Primer sets and multiplex conditions for detection of grapevine leafroll-associated viruses at the species level.** All primers were designed by Osman et al. (2007, J. Virol. Methods 141: 22-29).

| GLRaV | Probe sequence (5’ to 3’) | Dye | Actual size (bp) | Plex | Concentration |
| --- | --- | --- | --- | --- | --- |
| 1 | F: ACC TGG TTG AAC GAG ATC GCTT | VIC | 168 | 1 | 400 nM |
|  | R: GTA AAC GGG TGT TCT TCA ATT CTC T |  |  |  |  |
|  |  |  |  |  |  |
| 2 | F: CAT TAT ATT CTT CAT GCC TCT CAG GAT | 6-FAM | 116 | 1 | 400 nM |
|  | R: GAT GAC AAC TTC TGT CCG CTA TAG C |  |  |  |  |
|  |  |  |  |  |  |
| 3 | F: NED-AAG TGC TCT AGT TAA GGT CAG GAG TGA | NED | 254 | 1 | 400 nM |
|  | R: GTA TTG GAC TAC CTT TCG GGA AAA T |  |  |  |  |
| 4 | F: ATA TAC ATA CCA ACC GTT GTG GGT ATA A | 6-FAM | 93 | 2 | 400 nM |
|  | R: CCC TAT AAA CTA GCA CAT CCT TCT CTA GT |  |  |  |  |
|  |  |  |  |  |  |
| 5 | F: AAC ACT CTG CTT TTC TGC TGG C | VIC | 162 | 2 | 400 nM |
|  | R: CTT TTT ATG TCC CGA TAA ACG AGT ACA |  |  |  |  |
|  |  |  |  |  |  |
| 9 | F: CGG CAT AAG AAA AGA TGG CAC | NED | 82 | 2 | 400 nM |
|  | R: TCT TTA TGT CAC GGT AGA CCA ACA C |  |  |  |  |
| 18 S | F: GTG ACG GAG AAT TAG GGT TCG | PET | 70 | 3 | 200 nM |
|  | R: CTG CCT TCC TTG GAT GTG GTA |  |  |  |  |

All reactions for virus detection were run in a 3-plex with the first reaction containing the GLRaV-1, GLRaV-2 and GLRaV-3, the second reaction with GLRaV-4, GLRaV-5 and GLRaV-9, and a final reaction with the host plant *18S rRNA* gene alone, which was used as a control.
