# Supplementary material for: Occurrence of Grapevine Leafroll-Associated Virus Complex in Napa Valley
Source: PLoS One. 2011 Oct 19;6(10):e26227. doi: 10.1371/journal.pone.0026227 (PMC3198396; doi:10.1371/journal.pone.0026227)
Supplement: Table S3 — Primer sets and multiplex conditions for detection of Grapevine leafroll-associated virus-3 at the variant level. Primer sets for the four variants diagnosed and the conserved coat protein gene (CP) primers were designed in this study but were based on work by Wang et al. (2011, Phytopathology 101: 445–450). Primer set for the internal control, 18 S, was designed by Osman et al. (2007, J. Virol. Methods 141: 22–29). (DOC) [file pone.0026227.s004.doc]

**Table S3. Primer sets and multiplex conditions for detection of *Grapevine leafroll-associated virus-3* at the variant level.** Primer sets for the four variants diagnosed and the conserved coat protein gene (CP) primers were designed in this study but were based on work by Wang et al. (2011, Phytopathology 101: 445-450). Primer set for the internal control, 18 S, was designed by Osman et al. (2007, J. Virol. Methods 141: 22-29).

| GLRaV-3 | Probe sequence (5’ to 3’) | Dye | Actual size (bp) | Plex | Concentration |
| --- | --- | --- | --- | --- | --- |
| 3a | F: GTC AGT GGT AAT AAG TTC TC | VIC | 119 | 1 | 500 nM |
|  | R: CCC GTT TGA AAA TTT GAC ACT |  |  |  |  |
|  |  |  |  |  |  |
| 3c | F: CTT ATA AGG TCG CTG AGG | NED | 174 | 1 | 500 nM |
|  | R: CGA TGG ACC TGT GCC GTC |  |  |  |  |
|  |  |  |  |  |  |
| 18 S | F: GTG ACG GAG AAT TAG GGT TCG | PET | 70 | 1 | 80 nM |
|  | R: CTG CCT TCC TTG GAT GTG GTA |  |  |  |  |
| 3b | F: TAT AGT CTG TAA GGC G | NED | 242 | 2 | 500 nM |
|  | R: AGG CTA GTT TCA AGG AAT CTG TGT |  |  |  |  |
|  |  |  |  |  |  |
| 3d | F: CTT GGG TGT TTA AGC GAT TAC | VIC | 287 | 2 | 500 nM |
|  | R: CTT TCT ACA ACG AAG AGT GAT TA |  |  |  |  |
|  |  |  |  |  |  |
| CP | F: GAA CTG AAA TTA GGG CAG ATA TA | 6-FAM | 320 | 2 | 500 nM |
|  | R: AAR AAC TTG TCT GGA TCY TT |  |  |  |  |

All reactions were run in a 3-plex with the first reaction containing the GLRaV-3a, GLRaV-3c and *18S rRNA* gene, while the second reaction contained GLRaV-3b, GLRaV-3d, and the CP primer sets. A final primer concentration of 500 nM was used for all primer sets, except the *18S* *rRNA*, which was run with 80 nM.
