# Supplementary material for: Occurrence of Grapevine Leafroll-Associated Virus Complex in Napa Valley
Source: PLoS One. 2011 Oct 19;6(10):e26227. doi: 10.1371/journal.pone.0026227 (PMC3198396; doi:10.1371/journal.pone.0026227)
Supplement: Table S4 — Primers used for sequencing of 428 bp of the coat protein gene of Grapevine leafroll-associated virus-3 isolates. (DOC) [file pone.0026227.s005.doc]

**Table S4. Primers used for sequencing of 428 bp of the coat protein gene of *Grapevine leafroll-associated virus-3*** isolates.

| PCR primers | Probe sequence (5’ to 3’) |
| --- | --- |
| CP 130F | F: GAA CTG AAA TTA GGG CAG ATA TA |
| CP 580R | R: GCC CAT AAC CTT CTT ACA CA |
| Sequencing Primers | Probe sequence (5’ to 3’) |
| CP 210F | F: TAG TAA GGC GAG TTT CTT A |
| CP 500R | R: GGC TCG TTA ATA ACT TTC GGT |
| LR3E-F1 | F: GAC GCCTGA AAC GGA GGT G |
| LR3E-F2 | F: TGG TAG AGA CGC CTG AAA CG |
| LR3F-R | R: ATC TGG CGC TGA AGT AGC C |

All isolates detected only by the CP primer set, except for one, generated reads when sequenced using primer CP 500R but not primer CP 210F. These isolates were subsequently labeled GLRaV-3e and for these samples, primer CP 130F instead of CP 210F was used to sequence in the forward direction. This sample set showed no variant-specific amplicons in the fragment analysis. The lone sample that generated a forward read when sequenced with primer 210F (isolate 43-15) was thought to be a mixed infection of two previously unidentified variants. Isolate 43-15 was subsequently labeled as GLRaV-3f and internal primers, labeled LR3E-F1, LR3E-F2 and LR3F-R, which were specific for GLRaV-3f and GLRaV-3e, were designed to specifically sequence them. The primers were designed by overlapping the forward read of 43-15’s sequence of GLRaV-3f with sequences from all GLRaV-3e isolates to find a region of low similarity between GLRaV-3f and GLRaV-3e (Table S4). A 428bp region from GLRaV-3f was generated and assembled using the same method as above. Additionally, to insure the results were not caused by our multiplexing approach, nine GLRaV-3e and one GLRaV-3f samples were re-run using the GLRaV-3 HSP70h and the CP primer set in separate reactions at 1000 nM concentrations per primer set. The samples were subsequently visualized in 2.0% agarose gel.
